# Has testing been normalized? An analysis of changes in barriers to HIV testing among men who have sex with men between 2000 and 2010 in Scotland, UK

**DOI:** 10.1111/j.1468-1293.2012.01041.x

**Published:** 2012-08-30

**Authors:** P Flowers, C Knussen, J Li, L McDaid

**Affiliations:** 1 Department of Psychology and Allied Health Sciences, School of Health and Life Sciences, Glasgow Caledonian UniversityGlasgow, UK; 2 MRC/CSO Social and Public Health Sciences UnitGlasgow, UK

**Keywords:** barriers to testing, gay men, HIV normalization, HIV testing

## Abstract

**Objectives:**

This paper examines changes in barriers to HIV testing amongst gay men. We compared data collected in 2000 and 2010 to assess changes in HIV testing behaviours, in community-level perceptions of barriers to HIV testing, and in the relative contributions of barrier measures.

**Methods:**

Cross-sectional surveys were conducted within the commercial gay scene in Glasgow with good response rates (78% and 62%) using a form of time and location sampling.

**Results:**

Major changes in HIV testing behaviours were observed between 2000 and 2010 (30.6% increase in testing within previous year). At the community level, the perceived benefits of testing [*t* (1284) = –8.46; *P* < 0.001] and the norm for HIV testing [*t* (1236) = –11.62; *P* < 0.001] increased; however, other perceived barriers did not change (fear of a positive result, clinic-related barriers and attitudes to sex with HIV-positive men). Multinomial logistic regression showed that fear of a positive test result remained a key barrier to HIV testing; however, a significant fear × year of survey interaction indicated that fear played a lesser role in differentiating those who had never been tested from those who had been tested in 2010 than it had in 2000.

**Conclusions:**

These findings suggest the partial normalization of HIV testing. While some barriers have reduced, other key barriers remain important. Interventions should be designed and evaluated that attend to both the biomedical and the psychosocial aspects of HIV testing (e.g. the meaning of positive test results, the sexual exclusion of positive men, and HIV-related stigma).

## Introduction

While across Europe HIV prevention policies differ 1, increasing both the number and frequency of HIV antibody tests amongst men who have sex with men (MSM) remains central to attempts to normalize HIV testing, reduce undiagnosed HIV infection, prevent new HIV infections and decrease HIV-related morbidity 2, 3. Treating HIV infection like any other infectious disease is paramount 4. HIV testing remains core to the clinical management of HIV infection 5–7; however, it now also figures as a core part of HIV prevention, reflected for example in recent constructs such as ‘treatment as prevention’ 8, other biomedical approaches to HIV prevention 9, 10, and indeed the range of older HIV testing-based risk-reduction strategies such as serosorting 11.

The shift in HIV testing policy in the UK from opt-in to opt-out testing 12 has been associated with unprecedented increases in testing at the community level 13, 14, a drop in undiagnosed HIV infection 15, and potentially, given the likely transferability of findings from the USA, a corresponding reduction in onwards transmission 16. The simple change in routine service provision has probably reduced many barriers to testing 13. It has removed, or at least diminished, the role of the HIV testing decision-making process at the level of the patient/client. In order to design and implement interventions to promote the normalization of HIV testing 17, it is essential that we identify and understand the remaining factors that appear to either promote or deter people from testing 18 and address barriers to regular, frequent testing 19. Recent publications have focused upon synthesizing the evidence concerning barriers to HIV testing 20–22. In a systematic review of barriers to testing within Europe, Deblonde *et al*. 21 considered barriers to testing at three distinct levels: the patient/client level, the health care provider level, and the institutional/policy level. At the level of the patient or client, the key barriers appear to be low risk perception, fear of HIV disease (and fear of the consequences of a positive test result), fear of HIV disclosure (including discrimination and rejection), and the accessibility of health services. In addition to these barriers, De Wit and Adam 20 identified the importance of the perceived benefits of testing, which they suggest are rarely concerned with antiretroviral therapy (ART). Equally, the importance of patient-related barriers such as lack of perceived risk, a lack of information regarding testing possibilities, stigmatization and fear of a positive test result have also been highlighted 22.

Consequently, it is important to examine how, across this period of change in levels of HIV testing, perceptions of barriers to testing have changed at the community level – thus identifying which factors remain perceived barriers. Equally, it is important to examine which factors are actually associated with HIV testing behaviour and how these have changed across time. The current paper examines five measures of barriers to HIV testing, using data collected from MSM in Scotland at two time-points: 2000 and 2010. We examine three research questions: the extent of change in HIV testing behaviours between 2000 and 2010; the extent to which community-level perceptions of barriers to testing have changed; and finally, the relative contributions of barrier measures (when controlling for differences between 2000 and 2010) in understanding differences between those tested recently (within the previous year), those tested over 1 year previously (‘nonrecent testers’) and those never tested.

## Methods

We conducted two cross-sectional surveys (in 2000 and 2010) in commercial gay venues within Scotland. The same time and location sampling strategy was used in both surveys 13, 23. In 2000, 803 valid responses were collected after approaching 1029 men (78%). In 2010, 822 valid responses were collected after approaching 1314 men (62%). To ensure comparability between samples, we excluded men who did not give a Scottish postcode when asked to indicate area of residence. We excluded respondents who indicated that they were HIV-positive from the current analysis as, once HIV has been diagnosed, further HIV testing ceases. The maximum sample sizes for the current analysis were 686 in 2000 and 696 in 2010. The wording of the key HIV-related questions was the same in both surveys. Ethical approval was granted by the Psychology Ethics Committee at Glasgow Caledonian University.

### Measures

Demographic variables included age and educational qualifications (degree or postgraduate *vs.* school/vocational). Sexual behaviour was assessed using the number of unprotected anal intercourse (UAI) partners reported in the year prior to data collection, categorized as 0, 1 or ≥ 2.

The dependent variable centred on HIV testing, specifically whether tested within the previous 6 months, 6–12 months previously, 1–5 years previously, over 5 years previously, or never tested. For additional analyses (see below) these categories were recoded to form three groups of men: recent testers (within the 12 months preceding data collection), nonrecent testers (tested at some point in their lives but not within the 12 months preceding data collection), and never testers (those who had never had an HIV test).

Five measures of barriers to testing were included in the current analysis 1: *perceived benefits of HIV testing*, which consisted of three items: ‘If more people had an HIV test there would be fewer new HIV infections’; ‘Having an HIV test can help you plan your life’; ‘Having an HIV test puts your mind at rest’ 2; *fear of a positive HIV test result*, which consisted of five items: ‘Fear of a positive result puts me off testing’; ‘I do not want to test because of the psychological consequences of a positive result’; ‘I would rather get ill than find out I was HIV positive’; ‘I would rather not know my HIV status than risk being told I am HIV positive’; ‘It's much better to live with an uncertain HIV status than waking up every morning actually knowing you're positive’ 3; *clinic-related barriers*, which consisted of four items: ‘Clinics don't open at the right times for gay men to get tested’; ‘The way staff treat people when they get tested puts them off having a test’; ‘Gay men avoid HIV testing because they can't bear waiting for the results’; ‘I wouldn't go for an HIV test if I had to wait more than a day for the results’ 4; *attitudes towards sex with HIV-positive partners*, which consisted of three items: ‘I wouldn't have anal sex with anyone I knew was HIV positive’; ‘I wouldn't have oral sex with anyone I knew was HIV positive’; ‘Nobody would want to have sex with me if they knew I was HIV positive’; and 5 *norm for HIV testing*, which consisted of one item: ‘Most of my gay friends have had an HIV test’. All items were rated on five-point scales from 1 (‘strongly disagree’) to 5 (‘strongly agree’). Scores were computed from the mean of contributing items.

### Analysis

The analysis was conducted using PASW Statistics 18.0 for Macintosh (SPSS Inc., Chicago, IL, USA). Scores for barrier measures were computed as means of contributing items, allowing for one missing value. Missing values on barrier items for both studies were imputed using the multiple imputation option in PASW Statistics 18.0 for Macintosh, provided that respondents had completed at least 50% of all barrier items. Differences between the two samples were investigated using χ^2^ analysis or *t*-tests, as appropriate. For the final analysis, multinomial logistic regression was used to examine the variables that accounted for variation in HIV testing behaviours. Variables were centred prior to inclusion and the contributions of interaction terms between year of survey and barriers to testing were examined after all main variables had been entered. Alpha was set at 0.05 (two-tailed). Where necessary, corrections were applied for inequalities of variance.

## Results

Descriptions of the two samples are presented in Table 1. The samples differed significantly on age distribution, such that in 2010 a higher proportion of the sample were aged 45 years or over, and a lower proportion were aged 25 to 34 years [χ^2^ (3, *n* = 1358) = 12.47; *P* = 0.006]. Overall, those sampled in 2010 were older than those sampled in 2000. There were no differences in terms of educational qualifications. With regard to sexual behaviour, the 2010 respondents were more likely to indicate having had two or more UAI partners, and less likely to have had no such partners, than were the respondents sampled in 2000 [χ^2^ (2, *n* = 1322) = 14.62; *P* = 0.001].

**Table 1:** Descriptive statistics for the 2000 (*n* = 686) and 2010 (*n* = 696) samples: age, education, unprotected anal intercourse (UAI) partners and HIV testing

	2000		2010		
			
	*n*	%	*n*	%	Difference (*P*-value)
**Age**					0.006
≤ 24 years	178	26.7	198	28.7	
25–34 years	264	39.6	219	31.7	
35–44 years	169	25.3	187	27.1	
≥ 45 years	56	8.4	87	12.6	
**Educational qualifications**					0.914
Secondary/vocational	390	60.5	418	60.8	
Degree/postgraduate	255	39.5	270	39.2	
**UAI partners in previous year**					0.001
0	400	62.3	359	52.8	
1	184	28.7	225	33.1	
≥ 2	58	9.0	96	14.1	
HIV testing*					< 0.001
In previous 6 months	49	7.7	271	39.7	
In previous 6–12 months	121	18.9	120	17.6	
In previous 1–5 years	105	16.4	114	16.7	
Over 5 years previously	46	7.2	41	6.0	
Never tested	318	49.8	137	20.1	

* Categories collapsed as follows prior to analysis: tested ≤ 12 months previously; > 12 months previously; never tested.

As expected, the two samples differed significantly on HIV testing behaviour. The key difference between the samples lay in the proportions of those tested recently (≤ 12 months previously) compared with those who had never been tested [χ^2^ (2, *n* = 1322) = 157.83; *P* < 0.001]. Among those sampled in 2000, 26.6% (*n* = 170) had been tested ≤ 12 months previously, and 49.8% (*n* = 318) had never been tested. However, among those sampled in 2010, 57.2% (*n* = 391) had been tested ≤ 12 months previously, and 20.1% (*n* = 137) had never been tested.

Changes in the perceived barriers to HIV testing are shown in Table 2. Those sampled in 2010 gained higher scores on the measures of testing benefits and testing norm than did those sampled in 2000, indicative of greater perceived benefit and a stronger testing norm [*t* (1284) = –8.46; *P* < 0.001 and *t* (1236) = –11.62; *P* < 0.001, respectively]. No differences between the samples were observed in the scores on the measures of fear of a positive test result, clinic-related barriers and attitudes to sex with HIV-positive partners.

**Table 2:** Changes in perceived barriers to testing at the community level

	2000			2010			
			
	α	M	SD	α	M	SD	Difference (*P*-value)
Perceived benefits of HIV testing	0.59	3.65	0.81	0.50	4.02	0.77	< 0.001
Fear of HIV-positive test result	0.82	2.19	0.85	0.83	2.21	1.03	0.698
Clinic-related barriers	0.58	2.48	0.70	0.63	2.55	0.86	0.106
Attitudes to sex with HIV-positive partners	0.68	3.45	0.92	0.68	3.54	1.04	0.092
HIV testing norm		2.96	0.89		3.61	1.13	< 0.001

M, mean; SD, standard deviation.

The final research question addressed the relative contribution of factors to understanding differences between those who were categorized as recent testers, nonrecent testers and never testers. This was examined through multinomial regression analysis. The dependent variable was HIV testing behaviour (three categories), and the independent variables were year of survey, age (as a continuous variable), level of educational qualification, number of UAI partners reported in the previous year, and the five barriers to testing. As noted above, there were significant differences between the years of survey in age and number of UAI partners; further, HIV testing varied with age at both time-points, and with number of UAI partners and with education in 2000.

The number of respondents included in the multinomial logistic regression analysis was 1133. The likelihood ratio test was significant [χ^2^ (20) = 395.74; *P* < 0.001], while the Pearson goodness of fit test was not [χ^2^ (2242) = 2256.79; *P* = 0.409]: it therefore appeared that the model provided a reasonable fit of the data. The overall percentage correctly classified was 58.3%.

The results are shown in Table 3. When adjusted for year of survey, age, education and number of UAI partners, those who had never been tested were distinguished from those who were recent testers by greater fear of a positive HIV test result, by a weaker perceived norm for HIV testing, by more negative attitudes to sex with HIV-positive partners, and by weaker perceptions of the benefits of testing. Those who had never been tested were distinguished from nonrecent testers by greater fear of a positive test result, a weaker perceived norm, and more negative attitudes to sex with HIV-positive partners. Recent testers were distinguished from nonrecent testers only by fear of a positive HIV test result.

**Table 3:** Results of multinomial regression analysis of recency of HIV testing, with adjusted odds ratios (AORs), 95% confidence intervals (CIs), and *P* values

	Never tested *vs.* recent testers				Never tested *vs.* nonrecent testers				Nonrecent testers *vs.* recent testers			
	AOR	95% CI		*P*	AOR	95% CI		*P*	AOR	95% CI		*P*
Year of survey	4.86	3.46	6.84	< 0.001	2.26	1.57	3.27	< 0.001	2.15	1.52	3.03	< 0.001
Age	1.02	1.00	1.04	0.046	0.96	0.95	0.98	< 0.001	1.06	1.04	1.08	< 0.001
Education	1.25	0.90	1.73	0.175	1.53	1.08	2.16	0.017	0.82	0.60	1.13	0.227
UAI partners												
0	1.71	1.03	2.85	0.039	0.96	0.52	1.77	0.895	1.78	1.04	3.06	0.036
1	1.47	0.86	2.51	0.163	0.77	0.41	1.46	0.425	1.90	1.08	3.35	0.025
Fear of HIV-positive test result	2.19	1.76	2.71	< 0.001	1.53	1.22	1.93	< 0.001	1.42	1.14	1.78	0.002
Clinic-related barriers	1.19	0.93	1.51	0.169	1.20	0.92	1.56	0.174	0.99	0.77	1.26	0.922
Attitudes to sex with HIV-positive partners	1.24	1.04	1.48	0.019	1.35	1.11	1.63	0.002	0.92	0.78	1.09	0.330
Perceived benefits of HIV testing	0.75	0.60	0.93	0.010	0.92	0.73	1.16	0.459	0.82	0.65	1.02	0.073
HIV testing norm	0.57	0.48	0.67	< 0.001	0.64	0.53	0.77	< 0.001	0.89	0.76	1.05	0.160
Year × fear	0.71	0.58	0.88	0.001	0.73	0.59	0.89	0.002	0.98	0.79	1.23	0.877

The reference group for year is 2000, that for education is graduate/postgraduate, and that for unprotected anal intercourse (UAI) partners is ≥ 2.

It is also worth considering the contributions of the other factors. First, it was noteworthy that nonrecent testers were significantly older than both those who had never been tested and recent testers. They were also more likely to have a degree or postgraduate qualification than those who had never been tested. With regard to UAI partners, recent testers were more likely than either nonrecent testers or never testers to have had ≥ 2 UAI partners in the previous year.

In order to compare the roles of barriers between the two time-points in more detail, each year × barrier interaction term was added separately to the equation. Only year × fear made a significant contribution (see Table 3). This contribution centred on comparisons between those who had never been tested and both recent testers and nonrecent testers: the contribution of fear to the analysis was significantly smaller in 2010 than it had been in 2000, such that differences between never testers and other respondents on fear of a positive test result were smaller.

In summary, the results highlight differences between those who had never been tested and both recent and nonrecent testers on fear of a positive test result, the norm for testing and attitudes towards sex with HIV-positive partners, irrespective of the year of data collection. However, the role of fear of a positive test result changed over time, such that it made a smaller contribution to the explanation of variance in 2010 than it had done in 2000. The results also highlight the role of fear in differentiating between recent and nonrecent testers, irrespective of year of data collection.

## Discussion

Between 2000 and 2010, HIV testing in the 12 months preceding data collection increased dramatically from 26.6 to 57.2%. Moreover, testing in the preceding 6 months increased from 7.7 to 39.7%. Equally, the percentage of participants who had never been tested dropped from 49.8 to 20.1%. These changes over time were independent of both demographic factors and sexual behaviour. This represents a key change in HIV-related health behaviour akin to the profound changes in condom use noted during the 1980s within this population, and, as we have reported elsewhere, are coterminous with the policy change from opt-in to opt-out HIV testing in genitourinary medicine (GUM) clinics 13, 24. Here, we discuss why they suggest the normalization of HIV testing.

Before addressing this and the implications of this paper for policy development and further research, it is worth rehearsing the limitations of this work. It relies on cross-sectional research designs, examines samples of men recruited through the commercial gay scene only, and relies upon self-report data. It does not represent the evaluation of any specific intervention designed to increase testing nor does it focus upon the barriers to frequent testing. Moreover, the reliability of some of our measures is variable. However, the analysis reiterates major increases in HIV testing behaviour, it describes changes in perceived barriers to HIV testing at the community level, and it highlights the reduced role of some barriers to HIV testing and shows which ones remain important in explaining HIV testing behaviours.

The 2010 data, when compared to other European data, show that HIV testing rates now appear relatively high in Scotland 22. It is worth noting that within Scotland, in addition to the change in testing policy within clinics from opt-in to opt-out, there have recently been three mass media campaigns (2008–2010) which have all promoted both sexual health and HIV testing every 6 months amongst those at risk of acquiring HIV infections and sexually transmitted infections (STIs). Although this finding is suggestive of the effectiveness of such campaigns, it is worth noting that a recent systematic review of the international evidence highlights that there is limited evidence that multimedia social marketing campaigns can effectively promote HIV testing among MSM in developed countries 25. It is also possible that increases in recent HIV testing relate to increases in STI testing 26.

Significant differences were observed between the mean scores of the two samples relating to both the perceived benefits of HIV testing and the perceived norm of HIV testing. Given the observed changes in testing behaviour, it is perhaps unsurprising that there were significant changes in the perceptions of peer norms in relation to HIV testing. In fact, this change suggests that men are talking to each other about their HIV testing behaviours. However, this should not be confused with the issue of men talking to each other about their HIV status. Again it is interesting to speculate upon the role of the three social marketing campaigns which ran in Scotland, all with a clear focus upon promoting HIV testing. The fact that perceived benefits of testing have changed at the community level is perhaps indicative of the increased acceptability of HIV testing (note that the measure used here relates to psychological benefits rather than medical advances in HIV treatment, which have remained relatively constant). Together these findings are supportive of the idea of the normalization of testing in that there have been key changes to the ways the MSM community perceives HIV testing. The findings suggest that HIV testing is an acceptable and normative behaviour for this population.

Yet not all perceived barriers at the community level have reduced. Fear of a positive result (the perceptions of the meaning and consequences of HIV testing), clinic-related barriers (the opening times and waiting time associated with testing) and attitudes to sex with HIV-positive partners (the sexual exclusion of HIV-positive men by HIV-negative and untested men) showed no significant changes between data collection points. This suggests certain deficits within the normalization of HIV testing; some barriers to testing appear to endure despite an apparent shift in both behaviour and community norm. Despite the availability of testing through the opt-out policy, the success of antiretroviral therapy, and the reduced risk of transmission associated with undetectable viral load 27, HIV infection remains to some extent a stigmatized and dreaded condition 28.

The relevance of the perceptions at the community level is further illuminated by the results of the regression analysis. When controlling for time, age, educational attainment and HIV risk-related behaviour, fear of a positive test result remained important as a key barrier to testing. Equally, attitudes to sex with positive men remained an impediment to recent testing. Yet the significant fear × year of survey interaction term showed that the role of fear of a positive test result had decreased in differentiating between those who had never been tested and those who had; so, although there is evidence that its role is diminishing, fear of a positive test result remained particularly important in explaining differences in testing behaviours. Therefore, our findings suggest some disparity between biomedical and psychosocial understandings of HIV. HIV normalization is partial and not unidimensional. Much of what is proposed within the ideas of HIV treatment as prevention relies on a model of universal testing and the uptake of, and adherence to, antiretroviral treatments 8. This suggests that the salience of barriers to HIV testing will become more relevant to the full spectrum of HIV care. Maximizing testing opportunities and reducing barriers to regular testing will become a central component of HIV prevention and, as such, devising innovative and evidence-informed approaches to testing interventions is important.

The findings reported here reiterate the complexity of HIV testing and the need for multifaceted public health initiatives that address biomedical aspects of HIV testing and the psychological, social and cultural contexts of testing and its consequences. It seems likely that the key challenge for developing effective HIV testing interventions in a variety of contexts across Europe relates to addressing these approaches in tandem. Challenging the perceived sexual exclusion of HIV-positive men by rethinking transmission risk in relation to testing and treatment and the biomedical notion of infectivity at the community level could be one useful example. Along these lines, social marketing campaigns, for example, could highlight the reduced infectivity of HIV-positive men who are on treatment with undetectable viral loads, and simultaneously highlight the increased potential infectiousness of those who have not tested recently (yet might be highly infectious if they have recently seroconverted). In this way, lack of recent and regular testing would become associated with transmission risk (and concomitant stigma and sexual exclusion) rather than positive HIV status *per se*. Equally, in the context of opt-out testing, interactions with health care providers at testing sites offer an opportunity to explore in depth, and to challenge, men's perceived barriers to testing (such as fear of a positive result) through a range of readily available intensive brief interventions 29. Thus, in order to promote the normalization of testing as a core part of treatment as prevention (regular and frequent testing, timely diagnosis, early treatment initiation, good adherence and reduced infectivity), it is vital to address the meaning and significance of HIV within a variety of MSM communities (namely HIV-related stigma and challenging issues such as partner notification and HIV status disclosure).

A recent systematic review 30 highlighted the paucity of good-quality evidence relating to how best to promote HIV testing among MSM. It did, however, note the promise of particular approaches such as rapid testing and counselling in community settings and intensive peer counselling. When combined with our findings, this suggest that potential interventions aimed at addressing psychosocial aspects of HIV testing – both the perceived consequences of positive test results and negative attitudes to sex with HIV-positive men – and harnessing normative influence to increase the regularity and uptake of rapid tests within community settings may well be viable candidate interventions for further development and evaluation.
